# Will the Requirement by the US FDA to Simultaneously Co-Develop Companion Diagnostics (CDx) Delay the Approval of Receptor Tyrosine Kinase Inhibitors for RTK-Rearranged (*ROS1-, RET-, AXL-, PDGFR-α-, NTRK1-*) Non-Small Cell Lung Cancer Globally?

**DOI:** 10.3389/fonc.2014.00058

**Published:** 2014-04-01

**Authors:** Sai-Hong Ignatius Ou, Ross A. Soo, Akihito Kubo, Tomoya Kawaguchi, Myung-Ju Ahn

**Affiliations:** ^1^Chao Family Comprehensive Cancer Center, University of California Irvine School of Medicine, Orange, CA, USA; ^2^National University Health System and Cancer Science Institute of Singapore, Singapore; ^3^Aichi Medical University School of Medicine, Nagakute, Japan; ^4^National Hospital Organization Kinki-Chuo Chest Medical Center, Sakai City, Japan; ^5^Division of Hematology-Oncology, Department of Medicine, Samsung Medical Center, Sungkyunkwan University School of Medicine, Seoul, South Korea

**Keywords:** companion diagnostics, *ALK*-rearranged NSCLC, *ROS1*-rearranged NSCLC, *RET*-rearranged NSCLC, fluorescence *in situ* hybridization, immunohistochemistry, next generation sequencing, reverse transcription-polymerase chain reaction

## Abstract

The discovery of anaplastic lymphoma kinase (*ALK*) rearrangement in non-small cell lung cancer (NSCLC) in 2007 and the approval of crizotinib for the treatment of advanced *ALK*-rearranged NSCLC in 2011 represents a landmark in the development of targeted oncology therapy. The approval of crizotinib was accompanied simultaneously by the approval of the Vysis (Abbott Molecular) break-apart fluorescence *in situ* hybridization (FISH) test as the companion diagnostic (CDx) test to detect *ALK* rearrangement. Pfizer, the manufacturer of crizotinib, sponsored the screening of thousands of patients and the standardization of the *ALK* FISH test as part of the approval process for crizotinib, a first in class ALK inhibitor. Many pharmaceutical companies are now using the Food and Drug Administration (FDA)-approved *ALK* FISH assay to enroll patients onto trials for their own respective ALK inhibitors. In essence they are “piggybacking” on the FDA-approved *ALK* FISH assay without having to pay for the development of a CDx, nor screening for *ALK*-rearranged NSCLC patients in the protocols because screening for *ALK* rearrangement is now the standard of care in NSCLC after the approval of crizotinib. Since 2007, rearrangement in more receptor tyrosine kinases (RTKs) such as *ROS1, RET, AXL, PDGFR-α, and NTRK1* have been discovered in NSCLC but the incidence of each subtype of RTK-rearranged NSCLC is quite rare. Crizotinib has now demonstrated significant clinical activity in *ROS1*-rearranged NSCLC patients. Whether crizotinib will gain official FDA approval for use in *ROS1*-rearranged NSCLC, on the other hand, remains unclear as there is no test for *ROS1*-rearrangement currently being developed to support US FDA approval as a CDx. This may be due in part to the fact that the full cost associated with the development of a pre-market approved-approved CDx must be borne by the company seeking the first drug approval in a new indication. Given the low incidence of *ROS1*-rearrangement in NSCLC, and the availability of crizotinib in most countries, a more cost-effective way is for crizotinib to gain compendium listing for *ROS1*-rearranged NSCLC in treatment guidelines. However, without a formal indication from the FDA, a drug cannot be marketed for off label use, it is unlikely that payers public or private will routinely pay for molecular testing for *ROS1*-rearrangement in NSCLC let alone reimburse off label use of crizotinib. Similarly, several marketed tyrosine kinase inhibitors (TKIs) in the US (sorafenib, sunitinib, vandetanib, cabozantinib, regorafenib) are potent RET inhibitors *in vitro*. It does not make sense for any one pharmaceutical company to shoulder the full cost of developing a particular CDx for *RET*-rearranged NSCLC where, once approved, it may be used by other pharmaceutical companies to gain addition labeling approval for their own RET inhibitors. Thus, the requirement by the US FDA that a specific CDx have to be co-developed and standardized for each of the molecular subtype of NSCLC as part of the drug approval process, while prudent, may have the un-intended consequence of deterring clinical development of these TKIs in these very rare molecular subsets of NSCLC. While we all march to the drumbeat of precision cancer medicine, the stringent requirement of co-development CDx for each molecular subtype of solid tumor may inadvertently make this goal substantially more difficult to achieve.

## Introduction

Achieving personalized medicine is the “holy grail” in oncology. The approval of crizotinib in the US, an anaplastic lymphoma kinase (ALK)/ROS1/MET multi-targeted tyrosine kinase inhibitor (TKI), merely 4 years after the discovery of rearrangement in ALK in non-small cell lung cancer (NSCLC) represented a landmark in oncology drug development and a significant step toward the goal of personalized medicine in oncology (1). The approval of crizotinib was accompanied the simultaneous approval of the Vysis (Abbott Molecular) break-apart fluorescence *in situ* hybridization (FISH) companion diagnostics (CDx) assay by the US Food and Drug Administration (FDA) for the detection of *ALK* rearrangement in NSCLC. The success of crizotinib has shone a bright spotlight on the existence of molecular subsets of NSCLC and other epithelial malignancies that are driven by rearrangement in receptor tyrosine kinases (RTKs) and heralded the era of RTK rearrangement in solid tumor oncology. Since 2007 other RTK-rearrangements in NSCLC have been discovered (Table 1). Concurrently, various diagnostic tests besides FISH have been offered by major commercial diagnostic companies in the US to detect the different RTK-rearrangements. Given the rarity of RTK rearrangement in NSCLC and the requirement by US FDA to develop an analytically and clinically validated CDx for approval of TKIs against each RTK-rearranged molecular cohort, challenges abound in persuading many pharmaceutical companies to pursue a simultaneous registration strategy. We will review the lessons learned from the development of crizotinib for *ALK*-rearranged NSCLC where several second generation ALK inhibitors are in now development due to the existence of an FDA-approved CDx, the ongoing challenges in gaining additional FDA approval for crizotinib in the treatment of *ROS1*-rearranged NSCLC due to a lack of an approved CDx for *ROS1*-rearranged NSCLC, the immense challenges in gaining approval for any currently marketed TKI that are also potential RET TKI for the treatment of *RET*-rearranged NSCLC due to again the lack of an FDA-approved CDx for *RET* rearrangement (Table 2). Additionally, we will discuss whether the first FDA-approved CDx is the optimal CDx going forward given the inevitability of technology obsolescence coupled with the exponential gain in knowledge in the understanding of these subsets of molecularly defined NSCLC. Finally, we speculate that if the current challenges of co-CDx approval are not overcame how the development of precision cancer medicine may be impeded.

**Table 1:** **Characteristics of RTK rearrangement in NSCLC**.

RTK rearrangement	Year identified	Fusion partners	Estimate prevalence (%)	Methods of initial identification	Select reference
ALK	2007	EML4-, KIF5B-, KCL-, TFG-	~5–8	Tumor DNA transfection, Phospho-kinase activation	Ou et al. (1)
ROS1	2007	CD74-, SDC4-, SLC34A2-, TPM3-, FIG-, KDEL2-, CCDC6-, LRIG3-, ERZ-	~2	Phospho-kinase activation	Gainor and Shaw (2)
RET	2012	KIF5B-, CCDC6-, NOCA4-, TRIM33-	~2	FISH, NGS, WGS	Gainor and Shaw (2)
AXL	2012	MBIP-	NA	WGS	Seo et al. (3)
PDGFR-α	2012	SCAF11-	NA	WGS	Seo et al. (3)
NTRK1	2013	CD74-, MPRIP-	~3^a^	FISH, NGS	Vaishnavi et al. (4)

*^a^3.3% in ALK, ROS1, RET negative NSCLC*.

**Table 2:** **List of potential RET inhibitors potentially for the treatment of RET-rearranged NSCLC**.

Compound	Trade name	Manufacturer	*In vitro* kinase IC_50_ (nM) against RET	*In vitro* cellular kinase IC_50_ (nM) against RET	*In vitro* IC50 (nm) against RET V804 mutant	Other targets	Approved indications in the US	In clinical trial for RET-rearranged NSCLC	CDx used to detect *RET* rearrangement in NSCLC trials
Regorafenib (5)	Stivarga	Bayer	1.5	~10	NR	VEGFR1-3, KIT, RAF-1, BRAF, BRAFV600E, PDGFR-β	Treatment refractory colorectal adenocarcinoma	No	N/A
Ponatinib (6)	Iclusig	ARIAD	7	0.7–11	12	Bcr-abl, FGFR1-4,	TKI resistance CML or Ph + ALL	Yes^a^ NCT01813734	FISH, NGS
Cabozantinib (7)	Cometriq	Exelixis	5.2	27–85	>5000	VEGFR2, c-MET	Medullary thyroid cancer	Yes NCT01639508	FISH, NGS
Lenvatinib (E7080) (8)	N/A	Eisai	1.5	48 (CCDC6-RET)	NR	VEGFR1-3, FGFR1-3, PDGFR, c-kit	N/A	Yes NCT01877083	NGS
Sunitinib (6)	Sutent	Pfizer	30	40–164	55	PDGFR, VEGFRs, c-kit, FLT-3	RCC, GIST, unresectable/metastatic PNET	Yes NCT01829217	FISH, NGS
Sorefenib (9)	Nexaavar	Bayer	47	~20–50	55	Raf, PDGFR, VEGFR2, VEGFR3, c-kit,	HCC, RCC,	No	N/A
Vandetanib (10)	Caprelsa	AstraZeneca	100	NR	NR	VEGFR, EGFR	Medullary thyroid cancer	Yes NCT01823068	FISH

*^a^Currently on hold*.

*N/A, not applicable; NR, not reported*.

*PDGFR, platelet derived growth factor receptor; NGS, next generation sequencing; PNET, pancreatic neuroendocrine tumor; VEGFR, vascular endothelial growth factor receptor*.

## The Discovery of Receptor Tyrosine Kinase-rearranged (*ALK-, ROS1-, RET-, AXL-, PDGFR-α-, NTRK1-*) NSCLC

All the RTK-rearrangements identified in NSCLC occur in genes of the human RTK family, which consists of 58 members (11). The discovery of *ALK* rearrangement in NSCLC in 2007 was significant because prior to the discovery it was believed that gene fusions especially involving RTK rearrangement were believed to be rare in epithelial tumors (12). It is abundantly clear that each subtype of RTK-rearranged NSCLC is itself a heterogeneous disease made up many different (and yet to be discovered) fusion partners translocated to the same RTK (Table 1). The complexity within each molecular subtype of RTK-rearranged NSCLC have implications on the CDx. Ideally a CDx should be technically simple and/or be easily standardized, cost-effective, but also provide “forward-looking” information such as the exact fusion variant with at the exact breakpoint so that subtle differences among the various fusion variants within each molecular subtype of RTK-rearranged NSCLC can be elucidated.

Rearrangement of *ROS1* in NSCLC was discovered contemporaneously in 2007 by one of the two groups that discovered *ALK* rearrangement (13). *ROS1* shares extensive amino acid sequence homology with ALK in particular within the kinase domain making ROS1 a potential target for ALK inhibitors (14). Prior to 2007, *ROS1*-rearrangement was discovered in glioblastoma multiforme (15) and subsequently has been discovered in other major epithelial tumor types including gastric (16) and colorectal adenocarcinoma (17).

The *RET* (rearranged during transfection) proto-oncogene was first identified in 1985 through transfection of NIH3T3 cells with human lymphoma DNA (18). RET rearrangement has also been well characterized in thyroid cancer (19). Since 2012, multiple groups using various techniques published the rearrangement of RET in NSCLC with four identified fusion partners so far (*KIF5B- CCDC6-, NOCA4-, TRIM33-*) (2) (Table 1).

Rearrangement of the tropomyosin-related kinase gene (TRKA) was first biologically characterized in 1986 in a colorectal carcinoma patient (20), when tropomyosin was found to be fused to an unknown DNA sequence that likely codes for a transmembrane RTK (*TPM3-TRKA*) (20). The normal function of TRKA is the receptor for neurotrophins and is responsible for differentiation into subtypes of sensory neurons. TRKA has been renamed as neurotrophic tyrosine receptor kinase 1 (NTRK1) as it is one of three members of NTRK family (21). In 2013, rearrangement in NTRK1 was reported in NSCLC involving fusion partners with CD74 and MPRIP as fusion partners (*CD74-NTRK1, MPRIP-NTRK1*) (4). Screening a panel of NSCLC that are pan-negative for oncogenic driver mutations, they found 3 out of 91 (3.3%) were positive for *NTRK1* rearrangement. Cell-based and xenograft assays using NTRK1 inhibitors in NTRK1 transformed cells led to inhibition of cellular proliferation and tumor shrinkage, respectively, indicated *NTRK1* rearrangement are indeed a driver mutation in NSCLC (4). Of note similar to RET, rearrangement of *NTRK1* has been described in thyroid cancer (*TPM3-NTRK1, TPR-NTRK1, TFG-NTRK1*) (22).

*AXL*, termed from the Greek word anexelekto, or uncontrolled, was identified initially as a transforming oncogene in two chronic myelogeneous leukemia (CML) patients in 1991 (23). In 2012, *AXL* was found to be fused to MAP3K12 binding inhibitory protein 1 (MBIP) resulting in *AXL-MBIP* fusion variant by whole genome sequencing (WGS) (3). In the same study, Seo et al. also discovered the platelet derived growth factor receptor-alpha (*PDGFR-α*) was fused to SR-related CTD-associated factor 11 (*SCAF11-PDGFR-α*) in NSCLC (3). Prior to that, rearrangement in *PGDFR-α* was found in myeloid and lymphoid neoplasms with esinophilia where *PDGFR-α* is fused to Flip1-like 1 gene (FIP1L1) (*FIP1L1-PDGFR-α*) (24). Interesting aberrantly activation by phosphorylation of *PDGFR-α* was demonstrated in one cell line (H1703) and several patient samples in 2007 but no rearrangement was discovered (13). In summary, many of the RTK-rearrangements in NSCLC were discovered in other tumors but because of the success of crizotinib the discovery of these RTK-rearrangements in NSCLC has drawn increased attention to these RTKs in all tumor types (25).

## ALK Inhibitors for the Treatment of *ALK-* and *ROS1*-Rearranged NSCLC

While crizotinib is the first and only ALK inhibitor approved for the treatment of advanced *ALK*-rearranged NSCLC since August 2011, the majority of patients invariably progress on crizotinib with a median progression-free survival of about 8 months (26). The incorporation of break-apart ALK FISH as the FDA-approved CDx for detection of *ALK* rearrangement through the approval of crizotinib has provided a new standard of care with an established assay to screen for and enroll these *ALK*-rearranged NSCLC patients onto clinical trials of these ALK inhibitors. Pfizer, the manufacturer of crizotinib, engaged a diagnostic company to support both the development and technical validation of the *ALK* FISH CDx. In this case, Abbott Molecular sponsored the *ALK* FISH screening test and the validity of the CDx and the regulatory approval of the CDx as well as all screening of patients, to support the drug approval but Pfizer paid for everything Abbott Molecular. In retrospect, Pfizer essentially paved the way for competitors to more easily develop follow-on ALK inhibitors by establishing the clinical validity of a CDx test and screening for *ALK*-rearranged NSCLC patients. This realization, we believe has important implications on how the CDx for the other unique RTK-rearranged NSCLC may be developed by pharmaceutical companies.

Crizotinib has also shown significant clinical activity in *ROS1*-rearranged NSCLC due to the homology between the kinase domain (27). As part of the original phase I crizotinib trial (PROFILE1001, NCT00585195), the assay for the trial to detect *ROS1*-rearrangement is a locally developed laboratory-based test and no formal CDx is being developed for FDA approval in conjunction with the trial. In order for Pfizer to gain formal FDA approval for crizotinib in *ROS1*-rearranged NSCLC, Pfizer may have to sponsor another large scale trial and more importantly pay for the screening and analytical and clinical validation of a *ROS1* CDx (likely be FISH again) so that a CDx can be submitted simultaneously for FDA approval in support for the clinical activity of crizotinib in *ROS1*-rearranged NSCLC. However, once a CDx for *ROS1*-rearrangement is approved by the US FDA, other pharmaceutical companies can take advantage of the existence of an FDA-approved ROS1 CDx to develop their own ROS1 inhibitors similarly to the situations for current ALK inhibitors in clinical development. Given the low incidence of *ROS1*-rearranged NSCLC (~2%), Pfizer or other pharmaceutical companies is unlikely to make this investment given crizotinib is already available in many countries. Furthermore, although many Clinical Laboratory Improvement Amendments (CLIA)-certified commercial diagnostic companies in the US are offering *ROS1*-rearrangement testing [either by break-apart FISH, reverse transcription-polymerase chain reaction (RT-PCR), or even next generation sequencing (NGS)], without an official indication from the US FDA, screening for *ROS1*-rearrangement among community oncologists in the US will not be a common practice. Without an official FDA indication of crizotinib for *ROS1*-rearranged NSCLC, even with the endorsement of the National Comprehensive Cancer Centers Network (NCCN) guidelines, insurance companies may not pay for crizotinib for the few *ROS1*-positive NSCLC patients, even if their oncologists prescribe it. Furthermore, without an FDA indication for *ROS1*-rearranged NSCLC, the research of *ROS1*-rearrangement in other major epithelial tumor types such as colon (17) and gastric cancer (16), the cost of co-developing a companion diagnostics for *ROS1*-rearrangement will dissuade a lot of pharmaceutical companies to pursue a registration strategy in any *ROS1*-rearranged tumors even if they have potent ROS1 inhibitors in the pipeline.

## Will a RET Inhibitor Ever be Formally Approved by the US FDA for *RET*-Rearranged NSCLC and What is the Implication if the Answer is No?

We ask this question because the clinical reality of *RET*-rearranged NSCLC is even more relevant in illustrating the central theme of this perspective. There are currently at least six marketed TKIs (regorafenib, cabozantinib, ponatinib, sunitinib, sorafenib, vandetanib) in the US that are also potent *in vitro* RET inhibitors (Table 2). Under the current US FDA regulations, manufacturers of any one of the above marketed TKIs who wants to gain an additional approval for treatment of *RET*-rearranged NSCLC will have to pay for the screening for thousands of NSCLC patients and the development of a *RET*-rearrangement CDx. Again given the low incidence of *RET*-rearranged of NSCLC (~2%) and the potential crowded market for RET inhibitors, it is unlikely manufacturer of any one of the six potential marketed RET inhibitors will sponsor such as a trial, lest it will allow competitors to piggyback on the CDx to gain approval of their TKIs without shouldering the cost for patient screening and developing an approvable CDx. This is currently, the case as all the clinical trials in these marketed TKIs are investigator-initiated trials with a diverse platforms to screen for *RET* rearrangement (Table 2). Indeed, preliminary clinical activity of cabozantinib in three *RET*-rearranged NSCLC patients has been recently published (28). The exception is the manufacturer of lenvatinib (E7080) (Eisai Company, Ltd.) who is sponsoring a trial of lenvatinib in *RET*-rearranged NSLCL primarily in Asia using NGS as the primary CDx (NCT01829217) (Table 2). Without a US FDA-approved RET CDx, not only will potential RET inhibitors not gain US FDA approval to treat *RET*-rearranged NSCLC but other *RET*-rearranged malignancies such as thyroid cancer (19) or chronic myelomonocytic leukemia (CMML) (29).

Going forward, many small molecular inhibitors are being developed against *AXL* (30) and *NTRK1* (31, 32). Additionally, imatinib has shown excellent clinical activity against myeloid and lymphoid malignancies harboring *FIP1L1-PDGFR-α* rearrangement (33). Thus, achieving the goal of precision cancer medicine hinges on formal approval of these inhibitors to treat these various rare but diverse molecularly defined and driven malignancies and the requirement to co-develop a CDx may be a huge impediment to achieving this goal.

## Is the First Approved CDx the Best CDx Considering the Issues of Cost-Effectiveness, Knowledge Advancement, and Technology Obsolescence?

The approval of the Abbott Vysis break-apart FISH assay by the FDA as the CDx for the diagnosis of *ALK*-rearranged NSCLC seemed to have established break-apart FISH as the lead method platform to diagnose RTK rearrangement in NSCLC. However, break-apart FISH is probably “the worst of both worlds” as a CDx platform. There are three major criteria that have to be satisfied for a break-apart FISH to be considered positive: (1) a minimum of 50 cells have to be counted; (2) signals are considered “break-apart” when they are separated by at least two diameter in length OR only the 3′ signal is present; (3) at least 15% of the cells have to contain the break-apart signals. Polysomy is common in *ALK*-rearranged lung cancer tumor (34) thus, identifying all these criteria requires technical expertise and expert interpretation and is labor-intensive and time consuming. Additionally, FISH is prohibitively expensive as a mass screening method for many countries. Finally, FISH will not identify the specific fusion partner to the rearranged RTK gene. As our knowledge about RTK-rearranged NSCLC grows, it is highly likely that different RTK fusion variant will have different clinicopathologic characteristics such as extent of disease, site of metastasis, and differential response to TKIs (35), which required even more tailored treatment in the future. In summary, FISH is neither an inexpensive mass screening CDx nor does it lead to further understanding of the pathogenesis of RTK-rearranged NSCLC.

In contrast, ALK protein is only expressed in tumor tissue due to transcriptional activation from the promoter of the 5′-fusion partner to ALK but not in normal tissue and can be easily detected by immunohistochemistry (IHC). IHC is inexpensive and easily performed by all pathologists. Furthermore, ALK IHC has been demonstrated to show high concordance to ALK FISH (36). Since October 2012, IHC (Ventana automated staining system using D5F3 antibody from Cell Signaling Inc.) has been approved in the European Union (EU) as a CDx to detect *ALK* rearrangement along with break-apart FISH. This automated ALK IHC staining platform has shown extremely high sensitivity and specificity to ALK FISH (37). In September 2013 China approved the same method approved in EU to detect *ALK* rearrangement.

Immunohistochemistry has been used to detect *ROS1*-rearrangement in NSCLC and the sensitivity and specificity of ROS1 IHC is found to be 100 and 92%, respectively (38). Thus, it is likely with further refinement, IHC will likely be widely used to detect *ROS1*-rearrangement. On the other hand, RET is highly expressed in normal tissue and the sensitivity of RET IHC is low and thus, IHC may not be an ideal CDx to diagnose *RET* rearrangement (39). Thus, while IHC is a standard pathology procedure and cheaper than FISH, it is not applicable to all the different RTK-rearrangements depending on the normal expression pattern of the RTK in that particular tumor type. Much remain to be discovered on the expression level of *TRK1-, AXL-*, and *PDGFRα-* fusion proteins in NSCLC before we can really assess the utility of IHC in the detecting of these newly discovered molecular subtypes of RTK-rearranged NSCLC.

Reverse transcription-polymerase chain reaction is another commonly utilized research technique to detect RTK rearrangement. RT-PCR is highly specific and can be easily performed in standard diagnostic laboratories. However, most of the RT-PCR studies require large volume of tumor tissue snapped frozen from surgical resection. In daily oncology practice, the vast majority of the NSCLC are diagnosed from fine or core needle biopsy from which the tissue is placed in formalin instead of snap frozen at −80°C. RNA is not easily preserved in formalin-fixed tissues and thus RT-PCR may not be technically feasible in many of the samples. Also given that each unique molecular subtype of RTK-rearranged NSCLC has many different fusion variants; in order to identify all the known fusion variants the PCR has to contain primers to all the fusion partners. Any un-reported/un-discovered fusion partner will be missed by RT-PCR. In the case of *ROS1*-rearrangement, at least nine sets of primers for the nine reported fusion partners have to be present in the RT-PCR. Therefore, although RT-PCR has been commercialized in the US to detect RTK-rearranged NSCLC (40), it is not a widely adopted CDx and unlikely to gain global acceptance.

Next generation sequencing is a broad term that generally describes the massively parallel sequencing approach and employing various detection methods on a panel of genes that are altered in cancer. Many NGS panels of varying number of gene are now being offered commercially. For example, Foundation Medicine Inc., is offering a 236 gene test that can detect mutations, copy number alterations, and 19 gene rearrangements that has been used commercially used to detect new *RET* rearrangement in an investigator-initiated trial (28) or previously undetected *ALK* rearrangement (41).

Advances in the understanding of neoplastic diseases couple with technical advancement in the field of diagnostic tests raise the ongoing issue of technology obsolescence supporting the original FDA-approved test. Technology obsolescence will invariably poses a significant problem with time because one particular technology/diagnostic platform (i.e., FISH) is essentially linked to drug labeling by the FDA. With time that one specific diagnostic platform may turn out to be expensive, highly operator dependent with a steep learning curve, not easily automatable, and provide scant clinical information (e.g., FISH does not provide the fusion partner nor the break-point, which may be important in underlying the clinicopathologic and natural history of that particular RTK rearrangement). The ideal future CDx should be able to pinpoint chromosomal breakpoint and to identify the various fusion partners to a particular RTK rearrangement so that, we can continue to advance our molecular understanding of oncology in order to refine our approach to personalized medicine.

However, to get a different CDx platform approved by the FDA will again incur significant expense not only in standardization and validation of the new CDx but the cost of conducting a clinical trial “reinventing” the original approval process.

## Sample Survey of the Approved Indications for Crizotinib Outside the US

Crizotinib received conditional approval in the EU in July 2012 for previously treated ALK-positive NSCLC with the recommendation that a validated test for *ALK* rearrangement be used. Similarly crizotinib was approved in Singapore in 2013 for the treatment of locally advanced or metastatic *ALK*-rearranged NSCLC detected by an accurate and validated test. However, no one particular CDx (such as FISH) was specified by the approval in both EU and Singapore. Granted that in EU the approval of medicines and CDx are coordinated by two different agencies (42). Indeed, since October 2012, Vetana ALK IHC has been approved as a CDx for *ALK* rearrangement also. In Korea (2012), Japan (2012), and Australia (2013), crizotinib was approved for treatment of *ALK*-rearranged NSCLC without mention of the detection method. Granted by 2012, there is plentiful data supporting high concordance FISH and IHC (36) or even NGS (41) thus it is not necessary to pigeon-hole a drug approval to one particular CDx. However, without the initial US FDA approval of crizotinib and the advance in knowledge over the intervening years it is likely that “relaxed” CDx requirement will not be possible in many countries. Thus, approval of the US FDA remains the gold standard for the drug regulatory agencies and authorities in many countries.

## Concluding Perspectives

Many of the RTKs discussed in this perspective were discovered in 1980s as transformed oncogenes due to elegant basic science research. It has been more than 30 years since then to now where we are at the cusp of realizing precision cancer medicine by successfully translating these discoveries to therapeutic approvals and finally bearing fruit of all the research funding for the benefit of patients. The successful launch of crizotinib has been an inspiring example of this development.

The technologies to screen for these RTKs in all tumors are commercially available; inhibitors to these RTKs are either approved for other indication or in early clinical development. Because of the rarity of these RTK-rearrangements, the cost of sponsoring a registration trial for a particular TKI and simultaneous development of a CDx is prohibitively expensive and clinical progress is being delayed due to reluctance of pharmaceutical companies to pursue such narrow indications in rare disease populations.

One attractive though organizationally challenging solution may be to foster a collaboration of government, pharmaceutical companies, and diagnostic companies pooling resources together to an independent consortium to establish analytical and clinical validity of CDx platforms for detection of RTK-rearrangements and potentially other cancer genes. The US FDA may then approve these CDx platforms such as FISH, IHC, or NGS for each or several RTK-rearrangements and then allowing pharmaceutical companies to sponsor the trials and select any of the CDx platforms to demonstrate clinical benefit. This will alleviate the burden of simultaneously developing a CDx that can then be “piggybacked” by other pharmaceutical companies developing their own inhibitors. Additionally, this will eliminate potential conflict of interest as some global pharmaceutical companies also own major diagnostic companies (i.e., Ventana Medical Systems by F. Hoffmann-La Roche, Genoptix by Novartis) where one particular diagnostic platform may be favored by one pharmaceutical company due to technological knowhow and/or existing patents.

Short of industry-wide cooperation, regulatory policy may be used to lower regulatory burdens and create a more favorable incentive structure for therapeutic and diagnostics companies pursuing targeted therapy and CDx development. For instance, to reduce CDx costs, certain CDx quality systems and validation requirements may be simplified or deferred to the post-approval period, given appropriate risk determination. And as above, some assays may be approvable based on analytical validation data alone, decoupling diagnostic from therapeutic development decisions and thus streamlining coordination.

The requirement for co-development and co-approval of CDx in order to get TKIs approved against these RTK (*ROS1, RET, NTRK1, AXL, PDGFR-α*) rearrangement lung cancer represents the daunting challenge to successfully translate decades of basic science research into benefit of cancer patients. However, the successful approval of TKIs to treat *ROS1-, RET-, NTRK1-, PDGFR*-*α*, and *AXL-*rearranged NSCLC is vitally important as it sets the example for approval of TKIs to treat the same RTK-rearranged common epithelial tumors such as colon, gastric, and breast cancers (25). Using NSCLC as a tumor example, we wish this perspective contributed to the ongoing in-depth discussions about how to optimally and expeditiously develop TKIs to receive US FDA approval in the current regulatory environment where co-development and co-approval of a CDx is required for a drug in other TK-driven cancers.

## Conflict of Interest Statement

The authors declare that the research was conducted in the absence of any commercial or financial relationships that could be construed as a potential conflict of interest.
